# 

**Published:** 1910-03

**Authors:** 


MARCH, 1910.

Vol. XXVIII. No. 107.

LIBRARY Indexed S. U.

### THE BRISTOL MEDICO-CHIRURGICAL JOURNAL

LIBRARY SURGEON GENERAL'S OFFICE APR. -4. 1910

*PUBLISHED UNDER THE AUSPICES OF*

*THE BRISTOL MEDICO-CHIRURGICAL SOCIETY.*

---

*Editor:* R. SHINGLETON SMITH, M.D., B.Sc.

WITH WHOM ARE ASSOCIATED

J. MICHELL CLARKE, M.A., M.D.,

J. LACY FIRTH, M.S., M.D., JAMES SWAIN, M.S., M.D.

P. WATSON WILLIAMS, M.D., *Assistant Editor.*

*Editorial Secretary:* J. A. NIXON, B.A., M.B.

BRISTOL: J. W. ARROWSMITH.

LONDON: J. & A. CHURCHILL, 7 GREAT MARLBOROUGH STREET.

